# The Linear Programming to evaluate the performance of Oral Health in Primary Care

**DOI:** 10.1590/S1679-45082013000100017

**Published:** 2013

**Authors:** Claudia Flemming Colussi, Maria Cristina Marino Calvo, Sergio Fernando Torres de Freitas

**Affiliations:** 1 Universidade Federal de Santa Catarina, Florianópolis, SC, Brazil

**Keywords:** Health evaluation, Employee performance appraisal, Programming, Linear, Oral health, Primary Health Care

## Abstract

**Objective:**

To show the use of Linear Programming to evaluate the performance of Oral Health in Primary Care.

**Methods:**

This study used data from 19 municipalities of Santa Catarina city that participated of the state evaluation in 2009 and have more than 50,000 habitants. A total of 40 indicators were evaluated, calculated using the Microsoft Excel 2007, and converted to the interval [0, 1] in ascending order (one indicating the best situation and zero indicating the worst situation). Applying the Linear Programming technique municipalities were assessed and compared among them according to performance curve named “quality estimated frontier”. Municipalities included in the frontier were classified as excellent. Indicators were gathered, and became synthetic indicators.

**Results:**

The majority of municipalities not included in the quality frontier (values different of 1.0) had lower values than 0.5, indicating poor performance. The model applied to the municipalities of Santa Catarina city assessed municipal management and local priorities rather than the goals imposed by pre-defined parameters. In the final analysis three municipalities were included in the “perceived quality frontier”.

**Conclusion:**

The Linear Programming technique allowed to identify gaps that must be addressed by city managers to enhance actions taken. It also enabled to observe each municipal performance and compare results among similar municipalities.

## RESUMO

**Objetivo:**

Demonstrar o uso da Programação Linear na avaliação do desempenho da Saúde Bucal na Atenção Primária.

**Métodos:**

Foram utilizados os dados de 19 municípios catarinenses com mais de 50 mil habitantes que participaram de avaliação realizada no Estado em 2009. O modelo de avaliação utilizado era composto por 40 indicadores, que, depois de calculados em planilha eletrônica no *Microsoft Excel* 2007, foram convertidos para o intervalo [0,1], em ordem crescente (zero indicando a pior situação e 1, a melhor). Utilizando-se a técnica de Programação Linear, os municípios foram avaliados, de acordo com seu desempenho, em comparação aos demais municípios, a partir de uma curva de desempenho ótimo, denominada “fronteira de qualidade observada”, de modo que a qualidade dos municípios que estavam nessa fronteira foi considerada ótima, o que não ocorreu com os demais municípios. Os indicadores foram agregados, constituindo indicadores sintéticos.

**Resultados:**

Os municípios fora da fronteira de qualidade (valores diferentes de 1,0) ficaram, em sua maioria, com valores abaixo de 0,5, indicando baixo desempenho dos mesmos. O modelo aplicado aos municípios catarinenses avaliou a gestão municipal, o que implicou considerar as prioridades locais em detrimento de metas impostas por parâmetros pré-definidos. Na análise final da qualidade da Saúde Bucal na Atenção Primária, três municípios compuseram a “fronteira de qualidade observada”.

**Conclusão:**

A aplicação dessa ferramenta de análise possibilitou identificar os pontos nos quais os gestores municipais devem aperfeiçoar suas ações bem como observar seu desempenho relativo e compará-lo ao de municípios semelhantes.

## INTRODUCTION

The problem on how to use limited resources to achieve the most out of benefits, and the appearance of more complexes demands for the management of these resources make conventional approaches not enough to deal with performance evaluation. This scenario requires different tools capable of obtaining more satisfactory results by means of multidimensional analyses.

According to Rafaeli^(1)^ a model for performance evaluation must be able to identify ways to improve the performance of the evaluated units, besides monitoring the system through the inclusion of indicators. To identify comparable units with those already evaluated that could be used as reference in one or more requisite considered important in the evaluation (benchmarking) constitute one of the ways that could be used to achieve this improvement.

Linear Programming is a tool that has been used in studies of performance evaluation and can satisfy its conditions. This tool is a quantitative method to solve problems and to decide how to find some goals, like minimizing costs or maximizing benefits, subjected to limitations in the amount of required products or available resources. This tool may be used by managers to solve problems in the decision making process on placement of resources and other several activities within an organization. Because available resources are usually not enough to perform all activities in an excellent way, it is critical to find the solution for better distribution of resources that are going to be used. This solution is found by optimizing models^(2)^.

Recently the Linear Programming was applied in the health area as an option to analyze quantitative data. It showed great applicability in studies that involve management assessment. Despite the predominant use of this tool in studies of hospital efficiency^(3–5)^ its applicability in other analysis has been widely observed^(6)^. An example is the study by Salinas-Marinez et al.^(7)^ that evaluated the efficiency of primary care in actions on diabetes, and the research by Rabetti and Freitas^(8)^ that also evaluated efficiency of Primary Care in actions on hypertension.

## OBJECTIVE

To present the use of Linear Programming to evaluate the performance of Oral Health in Primary Care in municipalities of Santa Catarina State with more than 50,000 habitants.

## METHODS

In 2009 a study evaluating the quality of Oral Health in Primary Care was conducted in municipalities of Santa Catarina State. The model applied in the study was developed by the Extension and Research Center in Health Assessment (NEPAS, acronym in Portuguese)^(9)^.

This assessment model was composed by two dimensions: Oral Health management (management) and Oral Health basic care assurance (assurance). The first sub-dimensions included intersectoral action, popular participation, human resources and infrastructure, and the second focused on evaluation of children, teenagers, adults and elderly people considering actions of “diagnosis and treatment” and “promotion and prevention”. In management, four indicators were created for each sub-dimension (relevance, effectiveness, efficacy and efficiency), totalizing 16 indicators. In assurance, three indicators were created for each sub-dimension (relevance, effectiveness and efficacy), totalizing 24 indicators. In the NEPAS homepage (www.nepas.ufsc.br), which is available in Brazilian language, are the details of all 40 indicators, calculation methods and data source used.

A total of 207 municipalities were enrolled, but performance analysis using the Linear Programming was applied only to those with more than 50,000 habitants (19 municipalities) because they presented more stable indicators. So, the following methodologic procedures were applied to this group of municipalities.

A database was created by means of a Microsoft Excel 2007 spreadsheet program with the primary and secondary data related to the indicators for their calculation. The calculated indicators were converted to the interval [0, 1] in ascending order (1 indicating the best situation and zero indicating the worst situation).

Five and 95 percentiles were used as indicators for presenting outliers. Municipalities having values above or below these percentiles were converted to zero or one according to the positive or negative indicator variation.

The quality of municipal management was evaluated from a theoretical point of view, being the municipal management considered of quality when it presented “value” and “merit”. The evaluation was done in three steps: in the first step the value was defined from relevance measures and management efficacy; in the second step merit was defined from efficacy and efficiency measures; and in the third step value and merit measures generated quality measures.

Chart 1 shows these steps of indicator's aggregation to obtain the municipal quality values. Because the assurance dimension did not present efficiency indicators, the merit was constituted only by the efficacy indicators. To determine the final qualitymeasure, management and assurance quality measures were aggregated.

**Chart 1:** Indicators aggregation of relevance (I1), Effectiveness (I2), Efficacy (I3) and Efficiency (I4) to obtain value and merit measures, and quality of management and delivery and sub-dimensions

**Oral Health management**																							
**Intersection action**						**Public participation**						**Human resources**						**Infrastructure**					
I1	I2		I3	I4		I1	I2		I3	I4		I1	I2		I3	I4		I1	I2		I3	I4	
Value			Merit			Value			Merit			Value			Merit			Value			Merit		
AI Quality						PPOP Quality						HR Quality						IE Quality					
**Quality of Oral Health management**																											
**Delivery of Primary Care in Oral Health**																											
**Child**						**Teenager**						**Adult**						**Elderly**					
PP			DT			PP			DT			PP			DT			PP			DT		
I1	I2	I3	I1	I2	I3	I1	I2	I3	I1	I2	I3	I1	I2	I3	I1	I2	I3	I1	I2	I3	I1	I2	I3
V		M	V		M	V		M	V		M	V		M	V		M	V		M	V		M
Q_PP			Q_DT			Q_PP			Q_DT			Q_PP			Q_DT			Q_PP			Q_DT		
Q_CRI						Q_ADO						Q_ADU						Q_IDO					
**Quality delivery of Oral Health in Primary Care**																							

AI: intersection act; PPOP: public participation; HR: human resources; IE: infrastructure; Q_PP: promotion and prevention quality; Q_DT: diagnosis and treatment quality; Q_CRI: child quality; Q_ADO: teenager quality; Q_ADU: adult quality; Q_IDO; elderly quality; V: value; M: merit.

Representing this aggregation in a scatter chart, each point refers to value (V) and merit (M) measures of Oral Health management for each municipality (v, m). These measures are found in the interval [0, 1], being one given for the best situation and zero indicating the worst. So, the closest the point is to the origin the worst is its quality related to municipal management whereas as far it is from the origin the better the quality observed.

Municipalities were evaluated and compared among them using the Linear Programming technique according to performance, assuming that an excellent performance curve exists and it is delimitated by more distant points from the origin, corresponding to the best combinations (v, m). This curve is named “quality estimated frontier” and those included in this curve were considered excellent, in opposition to the other municipalities.

Applying Linear Programming the distance of each point from the quality estimated frontier is calculated in the interval [0, 1]. From this point on the municipality assumes higher values the more distant it is from the frontier, so indicating the worst performance. The municipalities that are in the frontier achieved the best performance and received a zero value. For a new aggregation the set of values is reorganized and converted again into a scale [0, 1] so that the worst performance (highest value, more distant from the frontier) assumes the zero value, and municipalities placed in the frontier (better performance) assume the 1 value.

Considering municipalities MUN=0,1,2,…n with values (v, m) for the characteristics V (value) and M (merit), being respectively 0≤v_n_≥1 and ≤m_n_≥1, the management of municipality 1was evaluated:

**GOOD**: when there is no municipality n for which [v_n_>v_1_ and m_n_≥m_1_] or [v_n_≥v_1_ and m_n_ >m_1_]

**BAD:** when there is some municipality n for which [v_n_>v_1_ and m_n_≥m_1_] or [v_n_≥v_1_ and m_n_>m_1_]

The following problem of Linear Programming was used: 
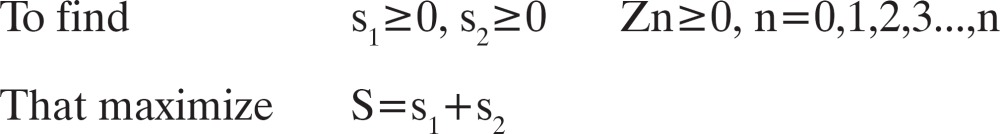



S represents the points (v, m) distance in relation to the quality estimated frontier.

This Linear Programming problem always has an optimal solution. So, management of a municipality can be considered excellent when it has S=0, since in this situation s_1_=s_2_=0. However, management can be considered bad when S= s_1_+s_2_>0 and if at least one of these values is positive.

Maximizing S=s_1_+s_2_ is equal to finding a point (V_n_, M_n_) more distant at the northeast point (v_1_, m_1_).

Linear Programming calculations were done using Microsoft excel Solver.

## RESULTS

From the aggregation of indicators, values for each municipality were obtained in a scale from zero to one, being one (highlighted in tables) given to municipalities that were included in “quality estimated frontier” in the respective dimension or sub-dimension. Municipalities distant from the frontier received a zero, indicating less quality.

Results of the municipalities' performance in all evaluated dimensions and sub-dimensions using the Linear Programming are shown on tables 1, 2 and 3. From the values on table 1 it is observed that despite sub-dimension “infrastructure” presented three municipalities in the quality frontier, this one had the worst mean among the management sub-dimensions. Municipalities not included in the quality frontier (values different from 1.0) were, in most cases, classified below 0.5, which indicated a bad performance.

**Table 1:** Municipalities performance in quality of management dimension and sub-dimensions from the aggregation indicators by the Linear Programming

Municipal	Quality				
	AI	PPOP	HR	IE	GSB
M1	0.635	0.866	0.766	0.686	0.763
M2	1,000	0.000	0.570	0.166	0.501
M3	0.797	0.237	0.810	0.143	0.357
M4	0.802	1,000	0.608	1,000	1,000
M5	0.000	0.105	0.673	0.371	0.000
M6	0.053	0.657	0.442	0.282	0.125
M7	0.693	0.330	1,000	0.748	0.681
M8	0.317	1.000	0.525	0.341	0.442
M9	0.680	0.720	0.649	0.449	0.573
M10	0.878	0.764	0.689	1,000	1,000
M11	0.411	0.272	0.460	0.733	0.308
M12	0.455	0.272	0.226	1,000	0.364
M13	1,000	0.221	0.609	0.418	0.736
M14	0.681	0.989	0.310	0.000	0.363
M15	0.829	0.559	0.735	0.494	0.637
M16	0.052	0.697	0.162	0.356	0.057
M17	0.867	0.847	0.496	0.187	0.594
M18	0.692	0.940	0.063	0.467	0.437
M19	0.682	0.738	0.000	0.000	0.128
Mean	0.606	0.590	0.515	0.465	0.477

Quality measures: AI: intersection act; PPOP: public participation; HR: human resources; IE: infrastructure; GSB: Oral Health management.

**Table 2:** Municipalities performance in quality of delivery dimension and sub-dimensions from the aggregation indicators by the Linear Programming

Municipal	Quality						
	CRI	ADO	ADU	IDO	PP	DT	PSB
M1	0.447	0.596	0.555	0.750	0.287	0.666	0.518
M2	0.002	0.163	0.460	0.620	0.007	0.287	0.177
M3	0.665	0.703	0.233	0.560	0.097	0.718	0.452
M4	0.606	0.547	0.288	0.611	0.285	0.517	0.421
M5	0.356	0.236	0.061	0.378	0.020	0.158	0.102
M6	0.344	0.378	0.000	0.000	0.008	0.000	0.000
M7	1,000	1,000	1,000	0.896	1,000	1,000	1,000
M8	1,000	0.758	0.667	0.691	0.931	0.626	0.753
M9	0.222	0.254	0.932	0.681	0.531	0.155	0.444
M10	0.840	0.628	0.552	0.992	0.759	0.709	0.838
M11	0.652	0.244	0.846	0.389	0.582	0.114	0.450
M12	0.522	0.935	0.151	0.279	0.595	0.223	0.362
M13	0.997	1,000	0.471	1,000	1,000	0.809	0.999
M14	0.060	0.694	0.951	0.591	0.480	0.338	0.506
M15	0.724	0.423	1,000	0.797	0.659	0.507	0.708
M16	0.241	0.668	0.414	0.555	0.246	0.473	0.369
M17	0.231	0.441	0.481	0.320	0.288	0.200	0.243
M18	0.000	0.000	0.388	0.445	0.000	0.060	0.048
M19	0.804	0.311	0.164	0.505	0.298	0.343	0.335
Mean	0.511	0.525	0.506	0.582	0.425	0.416	0.459

CRI: child quality; ADO: teenager quality; ADU: adult quality; IDO: elderly quality; PP; promotion and prevention quality; DT; diagnosis and treatment quality; PSB: quality of delivery of Oral Health.

**Table 3:** Quality of oral health service in Municipalities of Santa Catarina presenting more than 50,000 habitants in Management dimension and Assurance in ascending classification order toward final performance (FINAL)

Order	Municipal	Quality		
		GSB	PSB	Final
1^st^	M7	0.681	1,000	1,000
1^st^	M10	1,000	0.838	1,000
1^st^	M13	0.736	0.999	1,000
4^st^	M4	1,000	0.421	0.760
5^th^	M15	0.637	0.708	0.716
6^th^	M1	0.763	0.518	0.679
7^th^	M8	0.442	0.753	0.630
8^th^	M9	0.573	0.444	0.527
9^th^	M14	0.363	0.506	0.442
10^th^	M17	0.594	0.243	0.423
11^th^	M3	0.357	0.452	0.408
12^th^	M11	0.308	0.450	0.378
13^th^	M12	0.364	0.362	0.360
14^th^	M2	0.501	0.177	0.332
15^th^	M18	0.437	0.048	0.221
16^th^	M19	0.128	0.335	0.208
17^th^	M16	0.057	0.369	0.186
18^th^	M6	0.125	0.000	0.014
19^th^	M5	0.000	0.102	0.000
Mean		0.477	0.459	0.489

GSB: Oral Health management; PSB: quality of delivery of Oral Health.

Assurance dimension (Table 2) presented performance slightly higher in sub-dimension “promotion and prevention” (mean=0.425) than the sub-dimension “diagnostic and treatment” (mean=0.416).

From the data shown on table 3 management dimension had two municipalities on the “quality estimated frontier” (M4 and M10) whereas in assurance dimension only one municipality was seen (M7).

**Figure 1:**
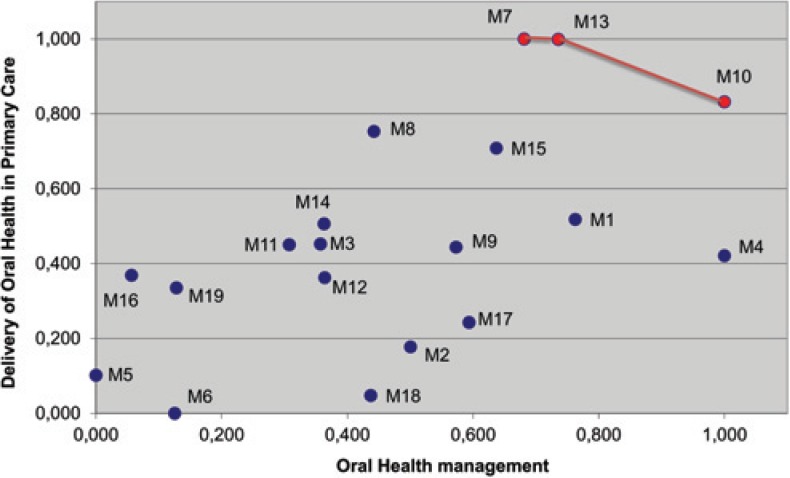
Dispersion chart of 19 evaluated municipalities, showing “quality estimated frontier” from analysis by the Linear Programming

Three municipalities were included in the “quality estimated frontier” in the last stage where final management and assurance values were aggregated. Figure 1 shows a scatter chart including municipalities M7, M13 and M10 that composed the frontier, being the more distant points from the origin responsible for better combinations between the two final measures. It is important to observe in the chart that none of these points had the value (1,1), which would mean an excellent performance in both dimensions.

## DISCUSSION

As stated in results sub-dimension “promotion and prevention” had better performance than “diagnostic and treatment”. The research by Lourenço et al.^(10)^ performed in Minas Gerais had an opposite result. They found that most actions were directed to clinical care. As for life cycles elderly health presented the highest mean, and adults health the worst. The assurance of Oral Care to children, which traditionally is a priority in odontology^(9)^, was not seen in such situation in the evaluated municipalities.

In this study the results reflect the characteristics of the indicators and measures used. Some measures of health prevention and promotion are linked to preventive programs commonly promoted by Oral Health services whereas measures related to assurance reflect more the curative actions impact, which are less traditional in odontology.

The evaluated municipalities had similar performance in assurance and management indicators, being observed a mild advantage of management indicators. Chaves and Vieira-da-Silva^(11)^ have worked with an assessment model structured in two dimensions named “Management Care of Oral Health” and “Practices of Oral Health”, however, in their study only two municipalities were evaluated and the performance towards the two dimensions was divergent.

These results show a real situation in which municipal managers were unable to achieve better results in all the aspects they were evaluated, that is, the prioritization of certain actions implies in the application of resources in detriment of resource restraint or lack of investment in other actions.

If municipality M7 is taken as an example, it is possible to note that its performance was weak in the management dimension, but it was good in almost all sub-dimensions of assurance, except in the item Oral Health in elderly people. These results suggest that during the studied period Primary Care in Oral Health in municipality M7 prioritized actions related to assurance and not management in agreement with the principles of *Sistema Único de Saúde* (SUS), which emphasis integrality and universality by performing actions that cover promotion and prevention as well as diagnostic and treatment for all ages^(12)^.

As for, municipality M10 that was included in the quality frontier in the final classification, together with municipalities M7 and M13, had a remarkable result in the management dimension.

Municipality M4 despite presenting an excellent performance in the management dimension was not included in the quality frontier because its performance in assurance was lower than municipality M10 that also had excellent performance in management. M4 efforts in both components were lower than those of the three other municipalities that composed the frontier.

Linear Programming analysis is different from the traditional score analysis because it enables to aggregate indicators, obtaining synthetic indicators that inform the performance in a specific dimension or sub-dimension in order to evaluate comparatively a set of analysis units (municipalities, in this case), which confers a multidimensionality. So, the “compensatory” effect is excluded from the sum of scores for the final quality value of municipalities that may occur when a municipality has a very bad performance on one dimension and a very good in another one, so that the good performance compensates the bad one, which usually leads to create an arbitrary classification with performance groups entitled as “good”, “intermediate” and “bad”. In the linear programming analysis it does not occur because those presenting the best arrangement are identified given the real conditions.

Another important difference towards traditional analysis of performance is the difficulty to state parameters for specific indicators. Not always theoretical stated parameters can be executed in practice, being ideal but not real.

An efficient indicator used in this assessment named “concentration procedures by concluded treatment” had as calculation form the total of procedures divided by the total of concluded treatments in the same period. The municipal Oral Care Programming of assistance based on coverage targets implies in the implementation of routine care based on completed treatments (CT) and not on free demand. To achieve efficacy using this care system it is necessary to consider the amount of procedures done until the patient finishes the treatment, being required to establish deadlines to the CT. Excluding the outliers, the values found vary from 1.0 to 8.6. In such case, what cut-off point should be adopted to state values related to municipal performance in this indicator? What value should be considered good or bad, satisfactory or unsatisfactory? Some parameters are at times well defined in the literature^(9–11)^. However, when this does not occur, as in this example, the appraiser must establish the cut-off point, which sometimes is arbitrary. Using the Linear Programming each municipal performance is compared with another similar municipality, so they are not compared using parameters.

The logic of analysis by Linear Programming is the same as the one used in the data envelopment analysis (DEA)^(5,6)^, however, this tool was not applied because no input and product variables were used. In fact, an aggregation of all indicators from the quality theoretical reference was performed.

In the logical evaluation of both the Linear Programming and the DEA the model proposed determined the place of each municipality in the quality ranking and established the benchmarking, that are municipalities in the quality frontier that work as parameters and motivators to other municipalities. However, these tools are not limited to the efficiency analysis and the relationship between input/product, opening up new possibilities for evaluations.

It is well know that evaluation of health actions in Brazilian municipalities has just started, and it is often restricted to measure result indicators. According to Veras and Vianna^(13)^, “ it is clear the need of including the evaluation process into the system management of health services in order to support the decision making process”. Therefore, the use and disclosure of analysis tools like the Linear Programming is critical to move forward in the process of evaluation institutionalization.

The analysis of Linear Programming results is based on the best practices or models to be achieved through the implementation of health policies, showing to managers the benchmarks and the pathways required to reach such results^(6)^.

The non-utilization of parameters or gold standard for each indicator is a methodological option in which the indicator performance is considered relatively to similar municipalities, even when the indicators have parameters established in the literature. Therefore, it is important to highlight that the model applied in the municipalities of Santa Catarina State pretended to evaluated municipal management, giving emphasis to local priorities rather than impos goals by pre-defined parameters.

This analysis tool also contributes to identify points in which municipal managers must improve their actions and observe their performance compared with similar municipalities. In addition, it is proposed as an option to DEA when the relationship between input/product is not used.

This study has chosen to present already aggregated data by sub-dimensions, however, the same analysis could be done from each indicator (relevance, effectivity, efficacy and efficiency) for each of the evaluated municipalities. To managers such disaggregated analysis provides additional information that could indicate actions that must be taken to improve global performance.

## CONCLUSION

The use of the Linear Programming to classify municipalities from the point of view of excellence in performance is a viable alternative of analysis that enables to identify points in which the municipal managers should improve actions and observe their municipal performance compared with other similar municipalities.
